# Application of Deep Convolutional Neural Networks for Discriminating Benign, Borderline, and Malignant Serous Ovarian Tumors From Ultrasound Images

**DOI:** 10.3389/fonc.2021.770683

**Published:** 2021-12-20

**Authors:** Huiquan Wang, Chunli Liu, Zhe Zhao, Chao Zhang, Xin Wang, Huiyang Li, Haixiao Wu, Xiaofeng Liu, Chunxiang Li, Lisha Qi, Wenjuan Ma

**Affiliations:** ^1^ School of Electrical and Electronic Engineering, TianGong University, Tianjin, China; ^2^ Tianjin Medical University Cancer Institute and Hospital, National Clinical Research Center for Cancer, Key Laboratory of Cancer Prevention and Therapy, Tianjin’s Clinical Research Center for Cancer, Tianjin, China; ^3^ The Sino-Russian Joint Research Center for Bone Metastasis in Malignant Tumor, Tianjin, China; ^4^ Department of Epidemiology and Biostatistics, West China School of Public Health, Sichuan University, Chengdu, China

**Keywords:** deep convolutional neural network, deep learning, ultrasound, serous ovarian tumor, transfer learning

## Abstract

**Objective:**

This study aimed to evaluate the performance of the deep convolutional neural network (DCNN) to discriminate between benign, borderline, and malignant serous ovarian tumors (SOTs) on ultrasound(US) images.

**Material and Methods:**

This retrospective study included 279 pathology-confirmed SOTs US images from 265 patients from March 2013 to December 2016. Two- and three-class classification task based on US images were proposed to classify benign, borderline, and malignant SOTs using a DCNN. The 2-class classification task was divided into two subtasks: benign vs. borderline & malignant (task A), borderline vs. malignant (task B). Five DCNN architectures, namely VGG16, GoogLeNet, ResNet34, MobileNet, and DenseNet, were trained and model performance before and after transfer learning was tested. Model performance was analyzed using accuracy, sensitivity, specificity, and the area under the receiver operating characteristic curve (AUC).

**Results:**

The best overall performance was for the ResNet34 model, which also achieved the better performance after transfer learning. When classifying benign and non-benign tumors, the AUC was 0.96, the sensitivity was 0.91, and the specificity was 0.91. When predicting malignancy and borderline tumors, the AUC was 0.91, the sensitivity was 0.98, and the specificity was 0.74. The model had an overall accuracy of 0.75 for in directly classifying the three categories of benign, malignant and borderline SOTs, and a sensitivity of 0.89 for malignant, which was better than the overall diagnostic accuracy of 0.67 and sensitivity of 0.75 for malignant of the senior ultrasonographer.

**Conclusion:**

DCNN model analysis of US images can provide complementary clinical diagnostic information and is thus a promising technique for effective differentiation of benign, borderline, and malignant SOTs.

## Introduction

Serous ovarian tumors comprise benign, borderline, and malignant lesions which have distinct clinicopathological characteristics, therapeutic schemes, and prognoses (1, 2). Accurate identification of SOTs prior to operation is critical to the development of appropriate treatments to avoid inadequate excision or surgical overtreatment (3). Pathology results revealed by fine-needle aspiration cytology are considered the gold standard for the diagnosis of SOTs before surgery or neoadjuvant chemotherapy. However, this method cannot be used to precisely identify the histological types of SOTs because of inadequate cytologic samples and the heterogeneous nature of the tissue composition (4).

Adnexal ultrasound is currently the first-line imaging modality for diagnosis of SOTs (5, 6). Although US imaging cannot replace biopsies, it can provide additional information that biopsies cannot deliver, such as intra-tumor heterogeneity. Diagnostic analysis of US images depends mainly on the physician’s expertise. However, in recent years, the emergence of artificial intelligence has brought hope for more objective and accurate diagnosis (7). Sakshi et al. (8) used a fine-tuned VGG-16 deep learning network in order to detect whether an ovarian cyst is present or not. Wu et al. (9) explored deep learning approaches for ovarian tumor classification based on ultrasound images. Zhang et al. (10) used an image diagnosis system for classifying the ovarian cysts in color ultrasound images.The application of deep convolutional neural network in medical image diagnosis has become a hot research topic (11–14). As of May 2020, more than 50 deep learning-based imaging applications have been approved by the US Food and Drug Administration or European Union, spanning most imaging modalities including X-ray, computerized tomography, magnetic resonance imaging, retinal optical coherence tomography, and ultrasound (15–19).

**Figure 1:**
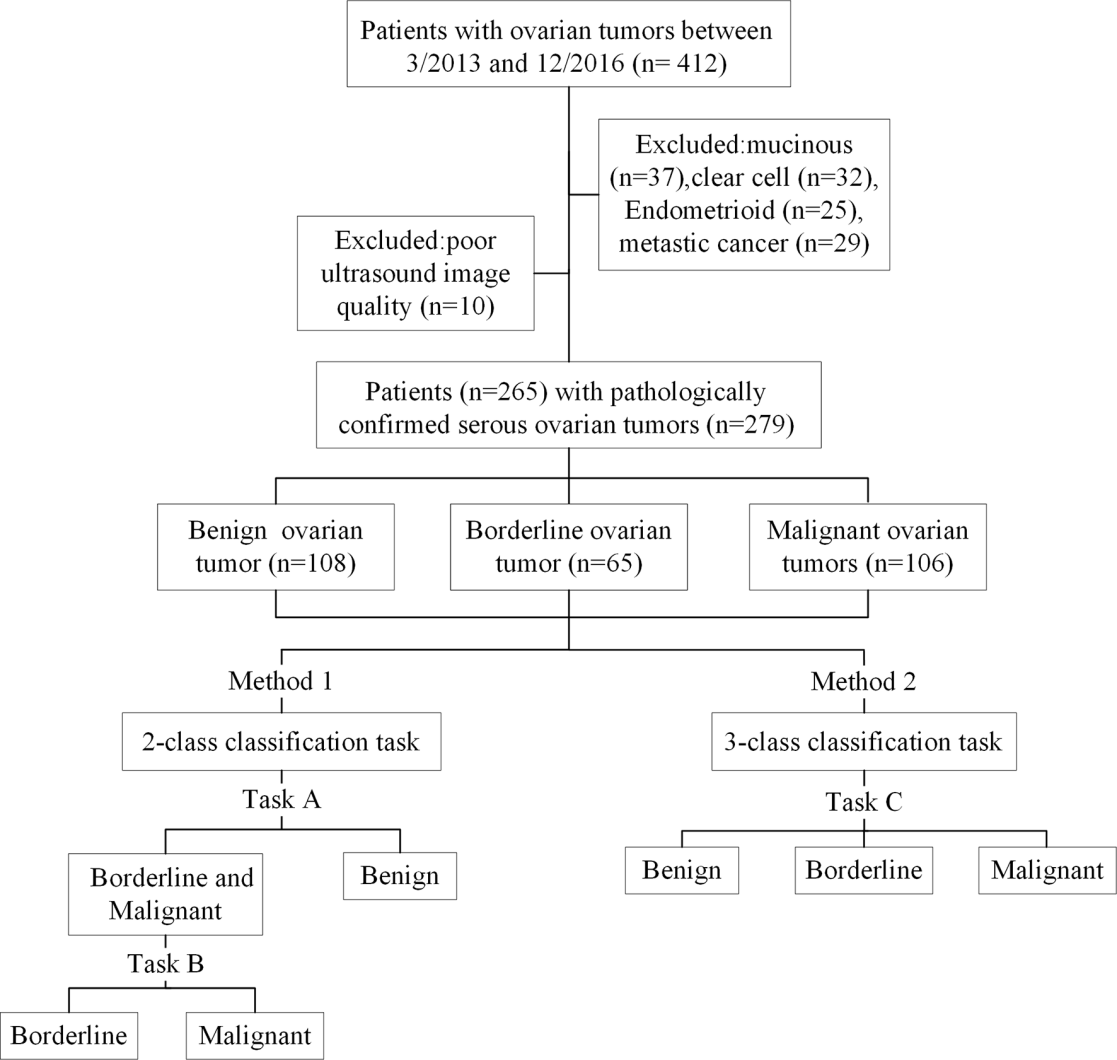
Flowchart of patient recruitment and the experimental design.

In the present study, we conducted 2- and 3-class classification task (**Figure 1**) using US images and deep learning methods to classify benign, borderline, and malignant SOTs. The results were compared with a senior sonographers with extensive diagnostic experience.

## Material and Methods

### Dataset

This study was approved by the Institute Review Board of Tianjin Medical University Cancer Hospital Institutional. Due to its retrospective nature, the informed consent requirement was waived.

**Figure 2:**
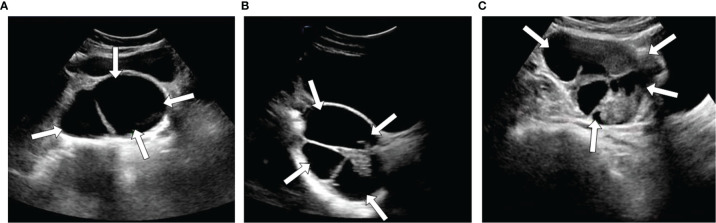
Three examples of ultrasound images with different types SOTs, benign **(A)**, borderline **(B)**, and malignant **(C)**.

A data set of US images in patients with SOTs from Tianjin Medical University Cancer Institute and Hospital(412 imaging studies, 265 patients)was collected. As shown in **Figure 1**, The patient inclusion criteria were as follows: (1) a histologic diagnosis of benign, borderline, or malignant SOTs between March 2013 and December 2016 (**Figure 2**); (2) availability of diagnostic-quality preoperative US images; and (3) US scanning before neoadjuvant therapy or surgical resection. The exclusion criteria were: (1) no ultrasound results or the ovarian mass was not completely in the images(10 images); and (2) mucinous(37 images), clear cell(32 images), endometrioid(25 images), or metastatic cancer(29 images). The included cases(279 images) were randomly assigned to either the training set (70%) or the validation set (30%).

US imaging was performed using equipment manufactured by Philips (EPIQ5, EPIQ7 and IU22), Samsung (RS80A), and GE Healthcare (LOGIQ E9, LOGIQ S7). The images were collected by transabdominal examination according to standard protocols and analyzed by ultrasound specialists.

### Data Preprocessing

All US images were retrieved from the Picture Archiving and Communication Systems for image segmentation and analysis in the hospital. Lesions were segmented using Image J software (https://imagej.nih.gov/ij/) by a sonographer with more than eight years of experience. All images were pre-processed: Due to the limited amount of training data, we used data augmentation techniques for image processing in order to avoid overfitting. The original images were randomly cropped and flipped using data enhancement technology, and all augmented images were resized to 224 * 224 pixels for input to the DCNN model. All pre-processing steps were conducted in Python (version 3.7.3; Python Software Foundation, Wilmington, Del) using the transforms imported from Torchvision (version 0.7.0).

### DCNN Model Training and Interpretation

At present, DCNNs are the most well-known type of deep learning architecture in the field of medical image analysis. Given their advantages, five representative DCNN architectures, namely VGG, GoogLeNet, ResNet, MobileNet, and DenseNet (20–24), were used to identify the histological types of SOTs based on US images. In the 2-class classification task, two tasks were trained and validated by the DCNN. The cohort with three classes was split into two-class datasets, and each sub-dataset was then evaluated by two-category classification (**Figure 1**). This yielded the following sub-datasets: benign *vs.* borderline & malignant (task A) and borderline *vs.* Malignant (task B). The 3-class classification task (task C)was used to directly identify benign, borderline, and malignant SOTs using the three-category classification DCNN.

During the training phase, the dropout strategy on the fully connected layers with a probability of 0.5 and L2 regularization strategy on weight and bias were used to prevent the overfitting problem, the initial learning rate was set to 0.0003 with a batch size of 32 and the Adam optimizer was used to update the weights of the neural network. All models were trained for 500 epochs; the learning rate is decayed every 20 epochs with a decay rate of 0.9. An Intel I7-9700K CPU and Nvidia GeForce RTX 2080 GPU were used for models training. In addition, we used the transfer learning methods of these networks for classification in order to compare the diagnostic efficiency of different methods (25–27). The weights of each network were initialized according to the weights from the pretrained model on ImageNet (28). We then fine-tuned the parameters of the fully connected layer of the network on our dataset *via* back propagation. The standard DCNN used comes from the Torchvision (version 0.7.0) package included in the PyTorch software framework. All programs were run in Python version 3.7.3.

To improve the interpretability of our model, we used the method of Class Activation Mapping (CAM) to visualize the important regions leading to the decision of the deep learning model (29). Such a localization map is completely generated by the fully trained network without additional manual annotation. By using a global average pooling layer and visualizing the weighted combination of the resulting feature maps at the penultimate (pre-softmax) layer, we obtained heat maps that explained what parts of an input US images were focused by the DCNN for assigning a diagnostic label. All heat maps were produced using the package OpenCV (version 4.3.0.36).

### Statistical Analysis

In the present study, we carried out 3-fold cross validation on different DCNN models, obtains the experimental results and calculates their indicators. The performance of the 2-class classification task was evaluated by the area under the receiver operating characteristic curve, accuracy, sensitivity, specificity, and F1 score. The performance of the 3-class classification task was evaluated by accuracy, sensitivity, and specificity only. Differences between the AUC values were considered statistically significant when *P* < 0.05. The method described by Hanley and McNeil was used to calculate the 95% confidence interval (CI) of the AUC values (30). These measurements were calculated using the numpy (version 1.16.2) Python library.

## Results

### Patient Characteristics

A total of 265 patients (median age 51 years, range 15–79 years) with 279 ovarian tumors (108 benign ovarian tumors, 65 borderline ovarian tumors, 106 malignant ovarian tumors), were retrospectively enrolled in this study.

### Performance of the Two-Class Classification Task

**Table 1** show the performance of the 2-class classification DCNN model using transfer learning and the full training methods on the validation sets. The AUC values of all models for the classification tasks were within the range of 0.827–0.963. In general, the transfer learning methods trained to distinguish benign from non-benign or borderline from malignant SOTs appear to perform better than the full training methods.

**Table 1:** Performance of the two-class classification deep convolutional neural network models in the validation set.

		AUC ( ± SD)	ACC ( ± SD)	SEN ( ± SD)	SPEC ( ± SD)	F1-Score ( ± SD)
**Transfer learning**						
**Task A**	VGG16	0.897 ( ± 0.016)	0.871 ( ± 0.018)	0.931 ( ± 0.015)	0.771 ( ± 0.023)	0.843 ( ± 0.019)
	GoogLeNet	0.924 ( ± 0.017)	0.883 ( ± 0.019)	0.828 ( ± 0.020)	0.972 ( ± 0.017)	0.894 ( ± 0.019)
	ResNet34	0.963 ( ± 0.016)	0.914 ( ± 0.017)	0.914 ( ± 0.015)	0.914 ( ± 0.018)	0.914 ( ± 0.017)
	MobileNet	0.885 ( ± 0.018)	0.871 ( ± 0.018)	0.931 ( ± 0.015)	0.771 ( ± 0.021)	0.843 ( ± 0.019)
	DenseNet	0.877 ( ± 0.019)	0.871 ( ± 0.021)	0.983 ( ± 0.016)	0.686 ( ± 0.022)	0.808 ( ± 0.019)
**Task B**	VGG16	0.865 ( ± 0.018)	0.882 ( ± 0.019)	0.944 ( ± 0.015)	0.714 ( ± 0.024)	0.813 ( ± 0.021)
	GoogLeNet	0.896 ( ± 0.018)	0.860 ( ± 0.019)	0.917 ( ± 0.016)	0.762 ( ± 0.023)	0.832 ( ± 0.020)
	ResNet34	0.914 ( ± 0.017)	0.893 ( ± 0.019)	0.983 ( ± 0.016)	0.743 ( ± 0.020)	0.846 ( ± 0.019)
	MobileNet	0.907 ( ± 0.017)	0.842 ( ± 0.021)	0.889 ( ± 0.019)	0.762 ( ± 0.021)	0.821 ( ± 0.020)
	DenseNet	0.898 ( ± 0.017)	0.825 ( ± 0.022)	0.806 ( ± 0.022)	0.857 ( ± 0.019)	0.831 ( ± 0.021)
**Full training**						
**Task A**	VGG16	0.886 ( ± 0.018)	0.839 ( ± 0.020)	0.931 ( ± 0.016)	0.686 ( ± 0.023)	0.790 ( ± 0.020)
	GoogLeNet	0.914 ( ± 0.017)	0.872 ( ± 0.019)	0.845 ( ± 0.022)	0.917 ( ± 0.017)	0.880 ( ± 0.019)
	ResNet34	0.909 ( ± 0.017)	0.893 ( ± 0.018)	0.966 ( ± 0.016)	0.771 ( ± 0.021)	0.858 ( ± 0.019)
	MobileNet	0.870 ( ± 0.018)	0.850 ( ± 0.019)	0.948 ( ± 0.016)	0.686 ( ± 0.024)	0.796 ( ± 0.021)
	DenseNet	0.900 ( ± 0.018)	0.850 ( ± 0.020)	0.966 ( ± 0.017)	0.657 ( ± 0.025)	0.782 ( ± 0.021)
**Task B**	VGG16	0.827 ( ± 0.021)	0.842 ( ± 0.021)	0.972 ( ± 0.016)	0.619 ( ± 0.025)	0.756 ( ± 0.022)
	GoogLeNet	0.843 ( ± 0.020)	0.842 ( ± 0.021)	0.999 ( ± 0.016)	0.571 ( ± 0.024)	0.727 ( ± 0.021)
	ResNet34	0.905 ( ± 0.018)	0.882 ( ± 0.019)	0.983 ( ± 0.015)	0.714 ( ± 0.023)	0.827 ( ± 0.020)
	MobileNet	0.845 ( ± 0.019)	0.825 ( ± 0.020)	0.917 ( ± 0.017)	0.667 ( ± 0.024)	0.772 ( ± 0.021)
	DenseNet	0.886 ( ± 0.018)	0.807 ( ± 0.020)	0.999 ( ± 0.017)	0.476 ( ± 0.025)	0.645 ( ± 0.023)

Task A: discriminating benign vs. borderline & malignant. Task B: discriminating borderline vs. Malignan.

AUC, area under the receiver operating characteristic curve; ACC, accuracy; SEN, sensitivity; SPEC, specificity; SD, standard deviation.

**Figure 3:**
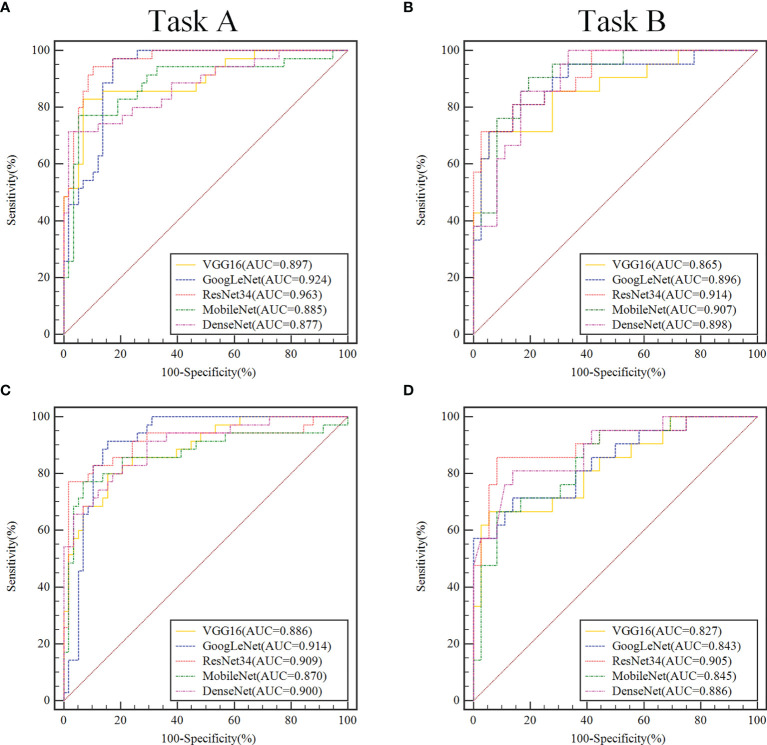
In the validation set, ROC curve analysis of two classification tasks with different convolutional neural network models before and after transfer learning. Task A **(A, C)** discriminating benign *vs*. borderline & malignant, Task B **(B, D)** discriminating borderline *vs*. malignant. In the convolutional neural network model, the models that use transfer learning are **(A, B)**, and the fully trained models are **(C, D)**.

When using migration learning methods for SOT classification in the validation sets of Task A (**Figure 3A**), the AUC values of VGG16, GoogLeNet, ResNet34, MobileNet and DenseNet models were more than 0.877( ± 0.019). When the full training method was used for differential diagnosis (**Figure 3C**), the AUC values of the above models were over than 0.870( ± 0.018). When using migration learning methods for SOT classification in the validation sets of Task B (**Figure 3B**), the AUC values of five DCNN models were more than 0.865( ± 0.018). When the full training method was used for differential diagnosis (**Figure 3D**), the AUC values of the above models were over than 0.843( ± 0.020).

In the validation sets, the ResNet34 models with the transfer learning method performs more comprehensively and better than other models in the 2-class classification task. When classifying benign and non-benign tumors, the AUC was 0.963( ± 0.016), the sensitivity was 0.914( ± 0.015), and the specificity was 0.914( ± 0.018), and the F1-Score was 0.914( ± 0.017). When predicting malignancy and borderline tumors, the AUC was 0.914( ± 0.017), the sensitivity was 0.983( ± 0.016), and the specificity was 0.743( ± 0.020), and the F1-Score was 0.846( ± 0.019).

### Performance of the Three-Class Classification Task

**Table 2** shows the performance of the 3-class classification DCNN model using transfer learning and the full training methods on the validation sets. The ResNet34 model using transfer learning methods exhibit higher discrimination performance than sonographer with 12 years of experience, with ACC values of 0.753( ± 0.019) and the sensitivity of this model to malignant tumors reached 0.889( ± 0.017). The sonographer’s overall diagnostic accuracy based on the semantic features of the images for differentiating benign, borderline, and malignant SOTs was 0.667( ± 0.021) and the sensitivity to malignant tumors reached 0.750( ± 0.018). Confusion matrix for 3-class classification DCNN modles are described in **Supplementary Figure 2**.

**Table 2:** Performance of the three-class classification deep convolutional neural network models and the senior sonographer in the validation set.

		Class	SEN ( ± SD)	SPEC ( ± SD)	ACC ( ± SD)
**Transfer learning**					
**Task C**	VGG16	Class 0	0.750 ( ± 0.022)	0.825 ( ± 0.018)	0.699 ( ± 0.021)
		Class 1	0.409 ( ± 0.025)	0.873 ( ± 0.017)	
		Class 2	0.829 ( ± 0.020)	0.845 ( ± 0.017)	
	GoogLeNet	Class 0	0.889 ( ± 0.016)	0.772 ( ± 0.021)	0.720 ( ± 0.020)
		Class 1	0.455 ( ± 0.024)	0.944 ( ± 0.016)	
		Class 2	0.800 ( ± 0.020)	0.897 ( ± 0.017)	
	ResNet34	Class 0	0.889 ( ± 0.017)	0.754 ( ± 0.022)	0.753 ( ± 0.019)
		Class 1	0.455 ( ± 0.024)	0.958 ( ± 0.015)	
		Class 2	0.800 ( ± 0.019)	0.897 ( ± 0.017)	
	MobileNet	Class 0	0.861 ( ± 0.018)	0.772 ( ± 0.021)	0.720 ( ± 0.020)
		Class 1	0.500 ( ± 0.023)	0.873 ( ± 0.017)	
		Class 2	0.714 ( ± 0.022)	0.931 ( ± 0.016)	
	DenseNet	Class 0	0.917 ( ± 0.017)	0.772 ( ± 0.021)	0.699 ( ± 0.023)
		Class 1	0.191 ( ± 0.027)	0.986 ( ± 0.019)	
		Class 2	0.857 ( ± 0.019)	0.759 ( ± 0.021)	
**Full training**					
**Task C**	VGG16	Class 0	0.917 ( ± 0.017)	0.632 ( ± 0.022)	0.667 ( ± 0.020)
		Class 1	0.917 ( ± 0.018)	0.772 ( ± 0.020)	
		Class 2	0.771 ( ± 0.019)	0.845 ( ± 0.021)	
	GoogLeNet	Class 0	0.944 ( ± 0.016)	0.684 ( ± 0.020)	0.710 ( ± 0.021)
		Class 1	0.500 ( ± 0.024)	0.901 ( ± 0.018)	
		Class 2	0.600 ( ± 0.023)	0.966 ( ± 0.018)	
	ResNet34	Class 0	0.778 ( ± 0.017)	0.825 ( ± 0.021)	0.710 ( ± 0.020)
		Class 1	0.500 ( ± 0.023)	0.845 ( ± 0.018)	
		Class 2	0.771 ( ± 0.017)	0.897 ( ± 0.022)	
	MobileNet	Class 0	0.861 ( ± 0.018)	0.719 ( ± 0.021)	0.688 ( ± 0.022)
		Class 1	0.409 ( ± 0.023)	0.916 ( ± 0.020)	
		Class 2	0.686 ( ± 0.021)	0.879 ( ± 0.020)	
	DenseNet	Class 0	0.889 ( ± 0.018)	0.737 ( ± 0.022)	0.677 ( ± 0.023)
		Class 1	0.318 ( ± 0.025)	0.901 ( ± 0.019)	
		Class 2	0.686 ( ± 0.020)	0.862 ( ± 0.020)	
**Doctor diagnoses**					
**Task C**	Doctor	Class 0	0.750 ( ± 0.018)	0.825 ( ± 0.022)	0.667 ( ± 0.021)
		Class 1	0.474 ( ± 0.023)	0.851 ( ± 0.019)	
		Class 2	0.684 ( ± 0.019)	0.818 ( ± 0.022)	

Task C: discriminating benign vs. borderline vs. malignant tumors.

Class 0: malignant tumors; Class 1: borderline tumors; Class 2: benign tumors.

ACC, accuracy; SEN, sensitivity; SPEC, specificity; SD, standard deviation.

### Visualizing and Understanding DCNN

**Figure 4:**
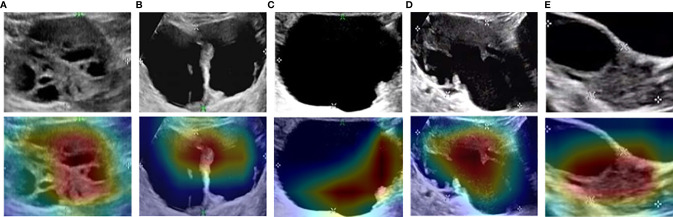
Examples of class activation mapping using the transfer learning ResNet34 model. The model correctly identified malignant **(A)**, borderline **(B)**, and benign **(C)** SOTs. Both the model and the senior sonographer made the same mistakes: borderline SOT misdiagnosed as malignant SOT **(D)**; and malignant SOT misdiagnosed as borderline SOT **(E)**.

As shown in the heatmaps produced using the means of the CAM results (**Figure 4**), Such a localization map is completely generated by the fully trained Restnet34 model without additional manual annotation.The red and yellow regions represent areas activated by the Restnet34 model and have the greatest predictive significance; the blue and green backgrounds reflect areas with weaker predictive values. The redder the feature color, the greater the possibility of the high DCNN score(**Figure 4A** DCNN score is 0.99, **Figure 4B** DCNN score is 0.71).We compared these findings with clinicians’ justifications. For the correctly diagnosed images (**Figures 4A–C**), the DCNN focused on the same areas as the clinicians. However, there were also some images that both the clinicians and DCNNs incorrectly diagnosed (**Figures 4D, E**). We also compared the areas of interest for the Advanced Sonographer and Resnet34, as detailed in the **Supplementary Figure 5**.

## Discussion

Predicting the specific histopathological condition from US images of patients with SOTs can help avoid unnecessary surgery on physiological hemorrhagic cysts. In this study using US images and a DCNN, we used 2- and 3-class classification task to distinguish benign, borderline, and malignant SOTs: 2-class classification task involved two task, task A distinguished benign vs. borderline vs. malignant tumors, and task B differentiated borderline vs. malignant tumors; 3-class classification task was a direct three-category classification. The results showed that the performance of the 2-class classification task was better than that of the 3-class classification task, although the process was more complicated. The use of transfer learning further improved the performance of the DCNN models.

In the present study, we also examined the capabilities of fine-tuning in comparison with a training scheme from scratch in the context of ultrasonic medical image analysis. Our experiments, which were based on five DCNN architectures, demonstrated that transfer learning DCNNs are useful for ultrasonic medical image analysis, performing as well as – and sometimes even outperforming – fully trained DCNNs. Another advantage of fine-tuned DCNNs is the training speed. To demonstrate this advantage, we compared the training times for a fine-tuned DCNN and a DCNN trained from scratch in **Table 3**. However, our results showed that transfer learning using a pre-trained model on ImageNet does not significantly improve the training of all DCNN models. Probably for this reason, ImageNet classes are images of animal, plants, and objects. Transfer learning may be better used for ultrasound image analysis if pre-trained models on medical image dataset are available in the future.

**Table 3:** The training time of transfer learning and full training method with epoch=500.

		VGG16	GoogLeNet	ResNet34	MobileNet	DenseNet
**Transfer learning**						
**Method1**	Step1	890	650	428	385	684
	Step2	584	431	281	259	684
**Method2**	Step1	912	654	436	387	690
**Full training**						
**Method1**	Step1	1791	626	707	567	1213
	Step2	1125	626	465	376	784
**Method2**	Step1	1776	638	720	581	1216

Unit of time: second.

To improve convincingness in our DCNN, we used “interpretable” method(CAM) that explains why they predict what they do. We also provided some heatmaps of the CAMs output, with visual examples of the explanation maps presented in **Figure 4**. Most SOTs are cystic-solid tumors. When identifying malignancy, doctors typically pay attention to the amount of solid area: the more solid components present in the US images, the higher the degree of malignancy. **Figures 3A, B** showed that the DCNN focuses on the solid components of the tumor, while **Figures 3D, E** showed cases in which both the DCNN and clinician made incorrect decisions in diagnosis. However, the imaging features of borderline and malignant SOTs greatly overlap. Therefore, it can be very difficult to distinguish between these two types of images. In future work, we plan to increase the training of cases to improve the accuracy of diagnosis. Furthermore, the prior knowledge of senior sonographers could also be integrated into the learning process.

Our research is subject to several limitations. Firstly, this was a retrospective study from a single center with a limited cohort size. External multi-center validation in a larger cohort is necessary to perform a better analysis. Secondly, since this was a retrospective study, there were cross marks left by the sonologist who made the diagnosis that could not be removed from the images. However, it can be seen from the CAM images that the DCNN did not pay attention to these cross marks, and they thus did not affect diagnosis (**Figure 3**). Thirdly, the performance reported for the different models may not be the best performance that can be achieved for each task, as performance is related to the hyper-parameters of DCNNs that influence the speed of convergence and final accuracy of the model. Identifying the optimal values for these hyper-parameters is rather difficult because each DCNN is a time-consuming process, even using high-end GPUs. Finally, US protocols and scanners, acquisition procedures, and parameter selection change over time, which may result in imaging variability and heterogeneity between patient cohorts.

## Conclusions

In conclusion, we demonstrated that DCNNs and transfer learning technology can achieve high accuracy at distinguishing benign, borderline, and malignant SOTs from ovarian ultrasound images. With further verification and model calibration in a larger cohort population, our DCNN-based model could be a promising tool for supporting clinical decision-making.

## Data Availability Statement

The data analyzed in this study is subject to the following licenses/restrictions: Due to the nature of this research, participants of this study did not agree for their data to be shared publicly, so supporting data is not available. Requests to access these datasets should be directed to Wenjuan Ma, mawenjuan@tmu.edu.cn.

## Ethics Statement

This study was approved by the Institute Review Board of Tianjin Medical University Cancer Hospital Institutional. Due to its retrospective nature, the informed consent requirement was waived.

## Author Contributions

WM, HQW, and CLL: study design and methods development. LQ and CXL: lesions identification and marking. HQW, CLL, and ZZ: results inference and implementation of methods. HQW, CLL, and CZ: manuscript writing. HQW, CLL, ZZ, XW, HL, HXW, XL, and WM: manuscript/results proof read and approval. All authors contributed to the article and approved the submitted version.

## Funding

This study has received funding by National Natural Science Foundation of China (82011530050, 81801781, 81903398, 82072004).

## Conflict of Interest

The authors declare that the research was conducted in the absence of any commercial or financial relationships that could be construed as a potential conflict of interest.

## Publisher’s Note

All claims expressed in this article are solely those of the authors and do not necessarily represent those of their affiliated organizations, or those of the publisher, the editors and the reviewers. Any product that may be evaluated in this article, or claim that may be made by its manufacturer, is not guaranteed or endorsed by the publisher.
