# Supplementary material for: Application of Deep Convolutional Neural Networks for Discriminating Benign, Borderline, and Malignant Serous Ovarian Tumors From Ultrasound Images
Source: Front Oncol. 2021 Dec 20;11:770683. doi: 10.3389/fonc.2021.770683 (PMC8720926; doi:10.3389/fonc.2021.770683)
Supplement: Supplementary file 1 [file DataSheet_1.docx]

**ELECTRONIC SUPPLEMENTARY MATERIAL**

**
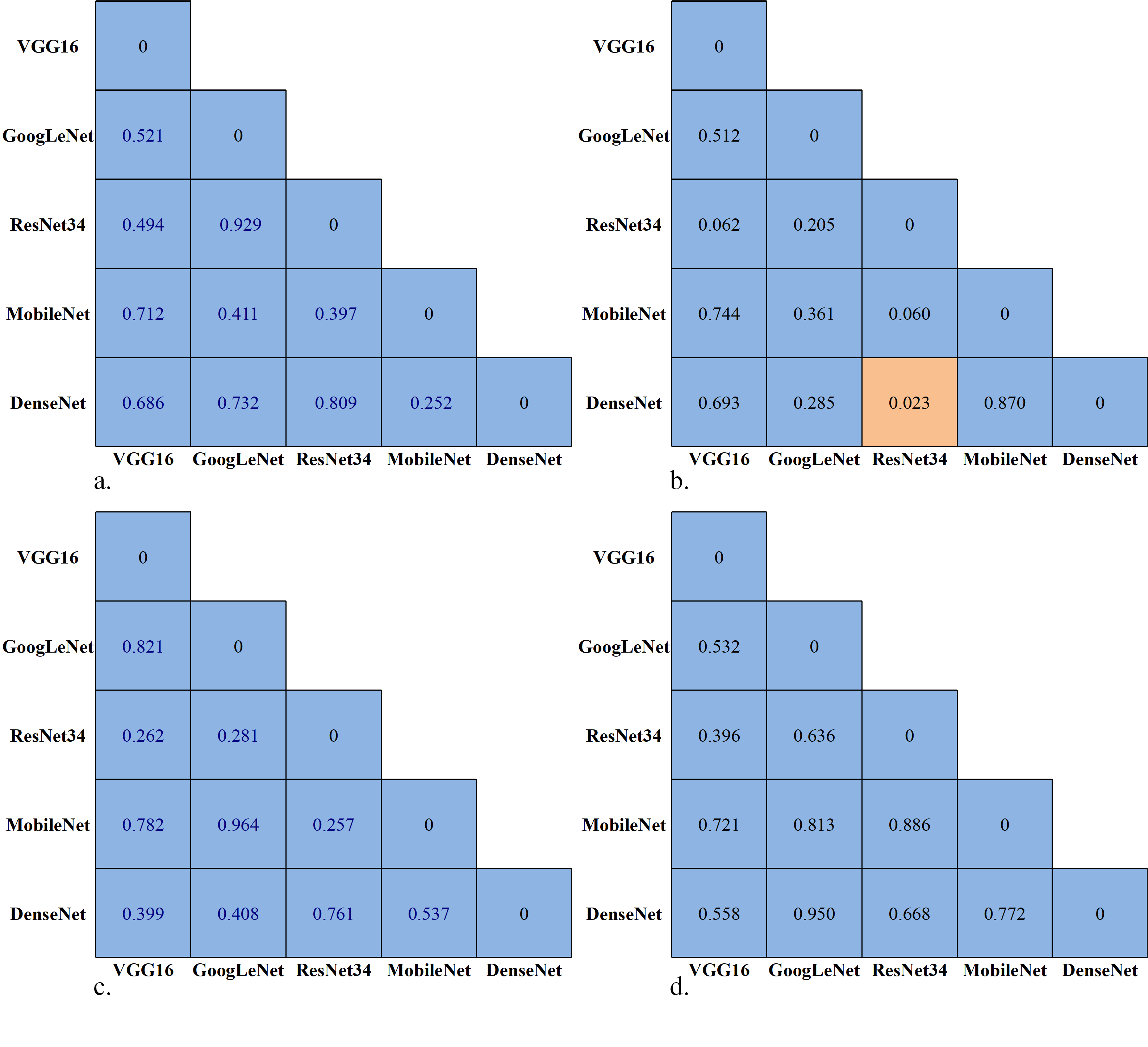
**

**Supplementary Figure 1.** In the validation set, the *P* value is used to show the statistical significance of the difference in AUC values between different models (blue for *P* ≥ 0.05 and orange for *P* < 0.05). Task A (a, c) discriminating benign *vs*. borderline & malignant, Task B (b, d) discriminating borderline *vs*. malignant. In the convolutional neural network model, the models that use transfer learning are a and b, and the fully trained models are c and d.


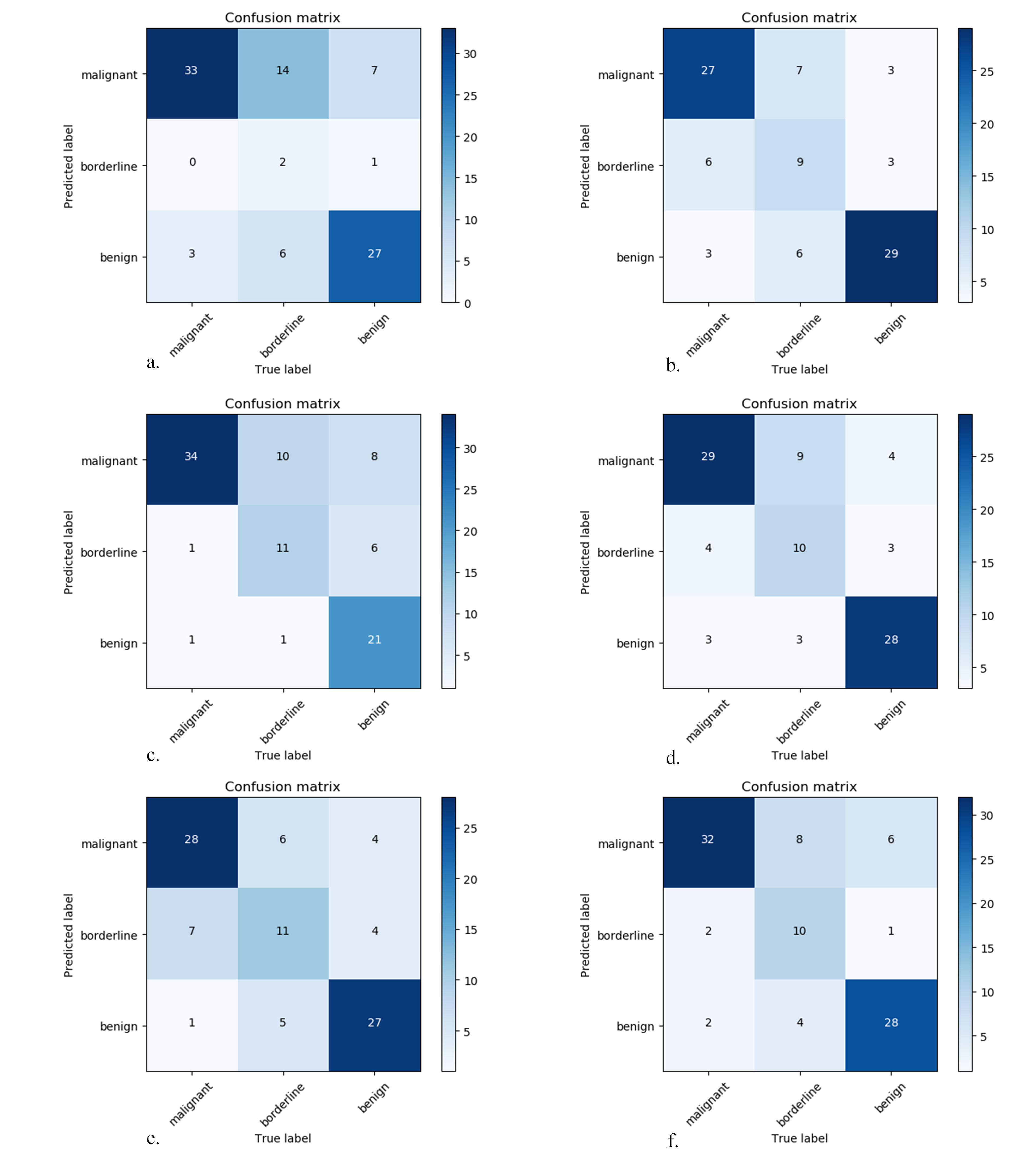


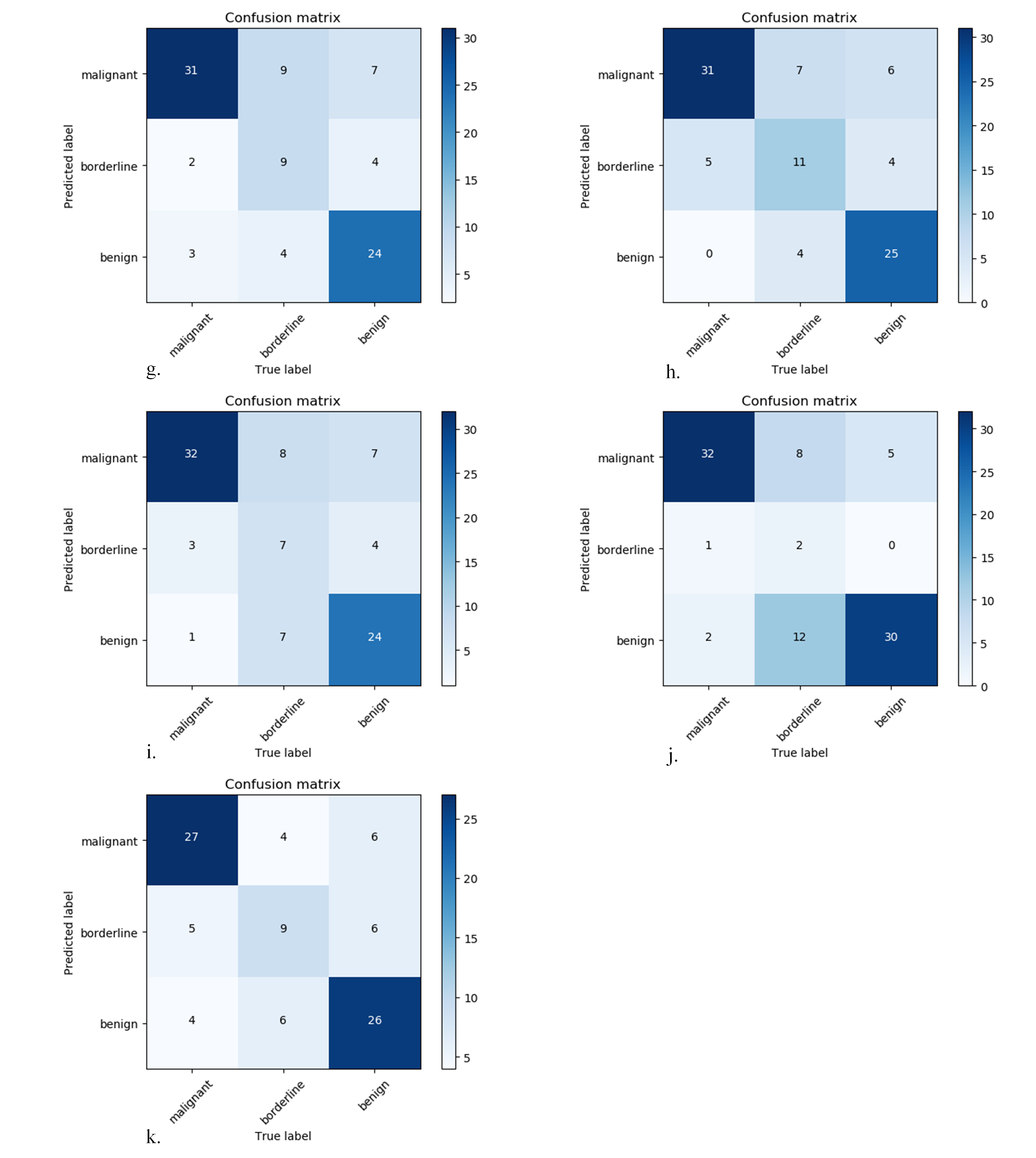


**Supplementary Figure 2.** In the validation set, Confusion matrix analysis of 3-class classification task with different convolutional neural network models before and after transfer learning. In the convolutional neural network model, the models that use transfer learning are VGG16 (b), GoogLeNet (d), ResNet34 (f), MobileNet (h), and DenseNet (j), and the fully trained models are VGG16 (a), GoogLeNet (c), ResNet34 (e), MobileNet (g), and DenseNet (i). Confusion matrix analysis of the performance of the sonographer on 3-class classification task (k).


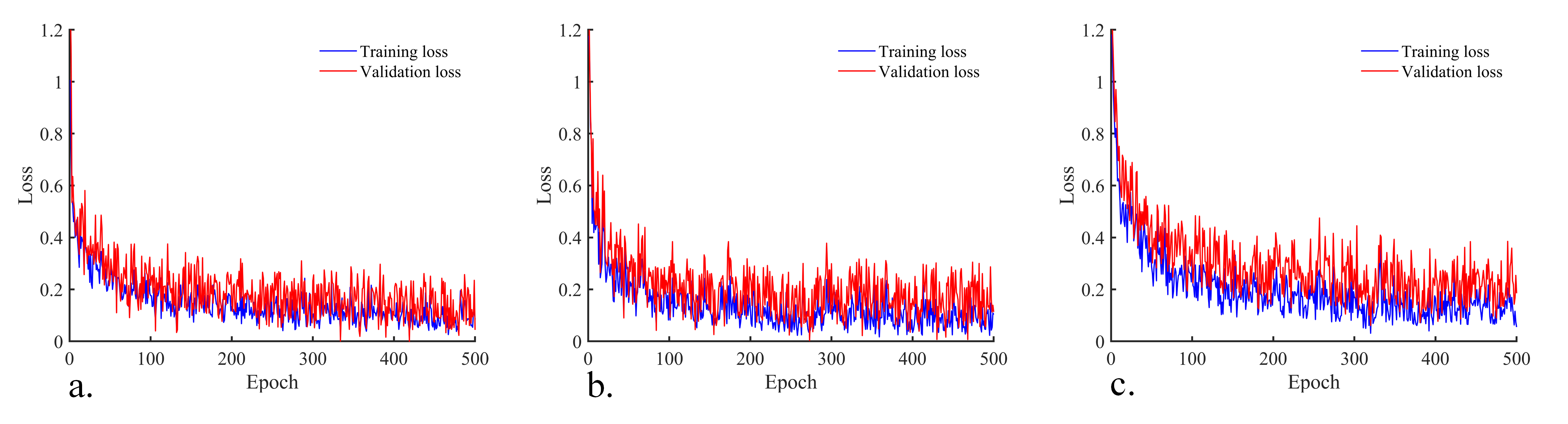


**Supplementary Figure 3.** The learning process of ResNet34 model for ovarian tumor classification using ultrasound images. The training loss and validation loss curves (blue and red lines) are plotted against the training process: Task A (a) discriminating benign vs. borderline & malignant, Task B (b) discriminating borderline vs. malignant, Task C (c) discriminating benign, borderline, and malignant.


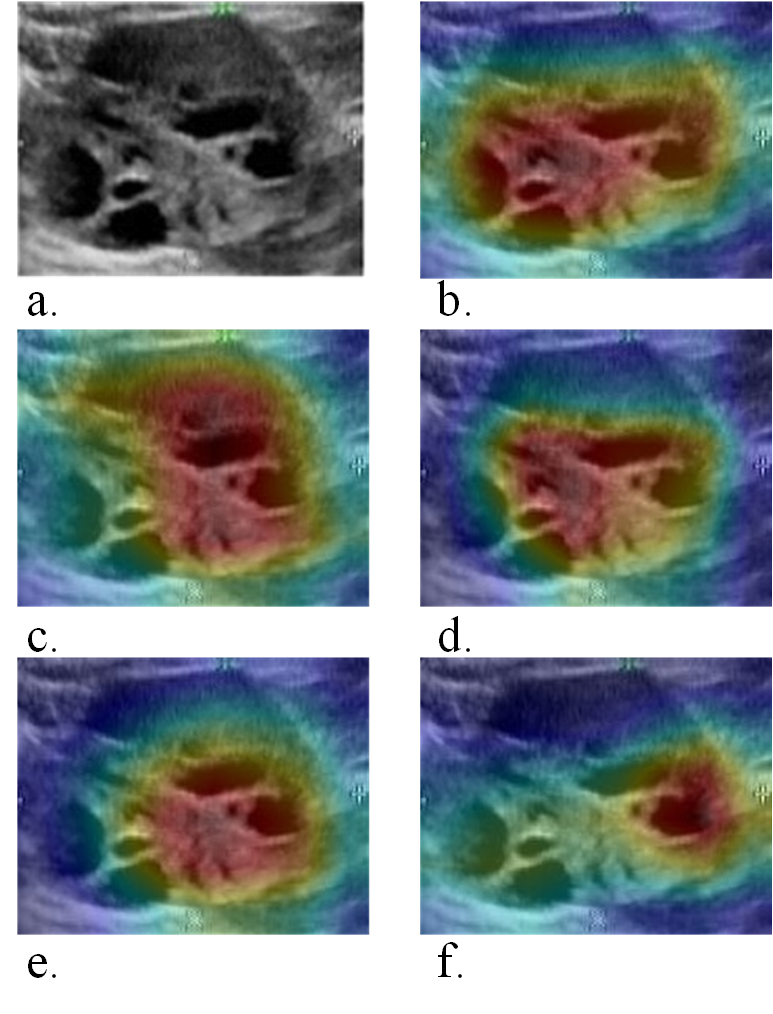


**Supplementary Figure 4.** Example of class activation mapping using transfer learning DCNN model. The original image is a malignant tumor (a) and we have demonstrated the results using five models, VGG16 (b), ResNet34 (c), GoogLeNet (d), MobileNet (e), and DenseNet (f), respectively.


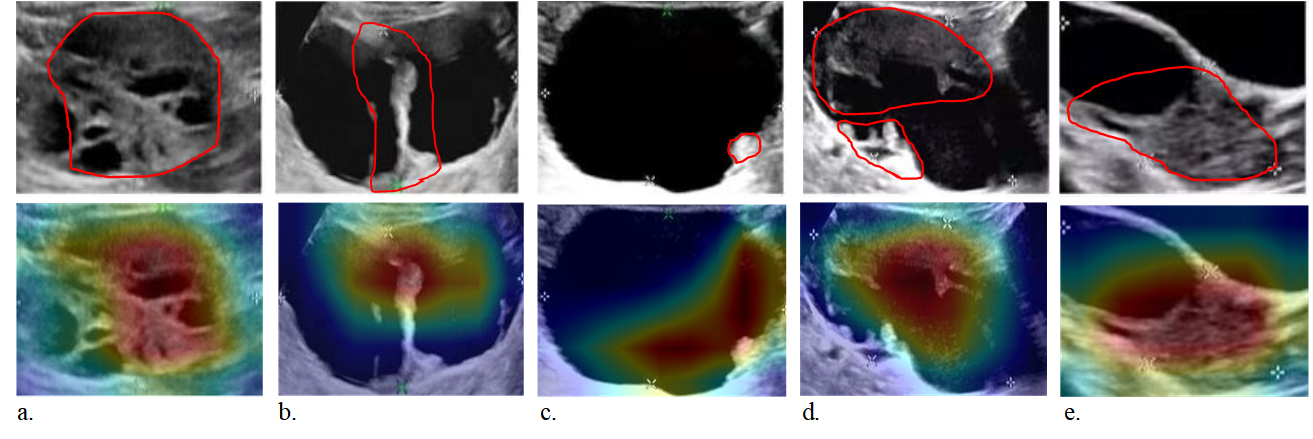


**Supplementary Figure 5.** Compares the areas of focus for the senior sonographer and Resnet34. The region of interest for the advanced sonographer is outlined by the red curve and the region of interest for resnet34 is shown by the CAM

**Supplementary Table 1.** Performance ranking of the two-class classification deep convolutional neural network models in the validation set.

|  |  | **AUC(±SD)** | **ACC(±SD)** | **SEN(±SD)** | **SPEC(±SD)** | **F1-Score(±SD)** |
| --- | --- | --- | --- | --- | --- | --- |
| **Transfer learning** | | | | | |  |
| **Task A** | ResNet34 | 0.963(±0.016) | 0.914(±0.017) | 0.914(±0.015) | 0.914(±0.018) | 0.914(±0.017) |
|  | GoogLeNet | 0.924(±0.017) | 0.883(±0.019) | 0.828(±0.020) | 0.972(±0.017) | 0.894(±0.019) |
|  | VGG16 | 0.897(±0.016) | 0.871(±0.018) | 0.931(±0.015) | 0.771(±0.023) | 0.843(±0.019) |
|  | MobileNet | 0.885(±0.018) | 0.871(±0.018) | 0.931(±0.015) | 0.771(±0.021) | 0.843(±0.019) |
|  | DenseNet | 0.877(±0.019) | 0.871(±0.021) | 0.983(±0.016) | 0.686(±0.022) | 0.808(±0.019) |
| **Task B** | ResNet34 | 0.914(±0.017) | 0.893(±0.019) | 0.983(±0.016) | 0.743(±0.02) | 0.846(±0.019) |
|  | MobileNet | 0.907(±0.017) | 0.842(±0.021) | 0.889(±0.019) | 0.762(±0.021) | 0.821(±0.020) |
|  | DenseNet | 0.898(±0.017) | 0.825(±0.022) | 0.806(±0.022) | 0.857(±0.019) | 0.831(±0.021) |
|  | GoogLeNet | 0.896(±0.018) | 0.860(±0.019) | 0.917(±0.016) | 0.762(±0.023) | 0.832(±0.020) |
|  | VGG16 | 0.865(±0.018) | 0.882(±0.019) | 0.944(±0.015) | 0.714(±0.024) | 0.813(±0.021) |
| **Full training** | | | | | |  |
| **Task A** | GoogLeNet | 0.914(±0.017) | 0.872(±0.019) | 0.845(±0.022) | 0.917(±0.017) | 0.880(±0.019) |
|  | ResNet34 | 0.909(±0.017) | 0.893(±0.018) | 0.966(±0.016) | 0.771(±0.021) | 0.858(±0.019) |
|  | DenseNet | 0.900(±0.018) | 0.850(±0.020) | 0.966(±0.017) | 0.657(±0.025) | 0.782(±0.021) |
|  | VGG16 | 0.886(±0.018) | 0.839(±0.020) | 0.931(±0.016) | 0.686(±0.023) | 0.790(±0.020) |
|  | MobileNet | 0.870(±0.018) | 0.850(±0.019) | 0.948(±0.016) | 0.686(±0.024) | 0.796(±0.021) |
| **Task B** | ResNet34 | 0.905(±0.018) | 0.882(±0.019) | 0.983(±0.015) | 0.714(±0.023) | 0.827(±0.020) |
|  | DenseNet | 0.886(±0.018) | 0.807(±0.020) | 0.999(±0.017) | 0.476(±0.025) | 0.645(±0.023) |
|  | MobileNet | 0.845(±0.019) | 0.825(±0.020) | 0.917(±0.017) | 0.667(±0.024) | 0.772(±0.021) |
|  | GoogLeNet | 0.843(±0.020) | 0.842(±0.021) | 0.999(±0.016) | 0.571(±0.024) | 0.727(±0.021) |
|  | VGG16 | 0.827(±0.021) | 0.842(±0.021) | 0.972(±0.016) | 0.619(±0.025) | 0.756(±0.022) |
| AUC area under the receiver operating characteristic curve, ACC accuracy, SEN sensitivity, SPEC specificity,  SD standard deviation  *Task A: discriminating benign *vs*. borderline & malignant.  *Task B: discriminating borderline *vs*. Malignan | | | | | | |

**Supplementary Table 2.** Performance ranking of the three-class classification deep convolutional neural network models and the senior sonographer in the validation set.

|  |  | **Class** | **SEN(±SD)** | **SPEC(±SD)** | **ACC(±SD)** | |
| --- | --- | --- | --- | --- | --- | --- |
| **Transfer learning** | | | | | | |
| **Task C** | ResNet34 | Class 0 | 0.889(±0.017) | 0.754(±0.022) | 0.753(±0.019) | |
|  |  | Class 1 | 0.455(±0.024) | 0.958(±0.015) |  | |
|  |  | Class 2 | 0.800(±0.019) | 0.897(±0.017) |  | |
|  | GoogLeNet | Class 0 | 0.889(±0.016) | 0.772(±0.021) | 0.720(±0.020) | |
|  |  | Class 1 | 0.455(±0.024) | 0.944(±0.016) |  | |
|  |  | Class 2 | 0.800(±0.020) | 0.897(±0.017) |  | |
|  | MobileNet | Class 0 | 0.861(±0.018) | 0.772(±0.021) | 0.720(±0.020) | |
|  |  | Class 1 | 0.500(±0.023) | 0.873(±0.017) |  | |
|  |  | Class 2 | 0.714(±0.022) | 0.931(±0.016) |  | |
|  | VGG16 | Class 0 | 0.750(±0.022) | 0.825(±0.018) | 0.699(±0.021) | |
|  |  | Class 1 | 0.409(±0.025) | 0.873(±0.017) |  | |
|  |  | Class 2 | 0.829(±0.020) | 0.845(±0.017) |  | |
|  | DenseNet | Class 0 | 0.917(±0.017) | 0.772(±0.021) | 0.699(±0.023) | |
|  |  | Class 1 | 0.191(±0.027) | 0.986(±0.019) |  | |
|  |  | Class 2 | 0.857(±0.019) | 0.759(±0.021) |  | |
| **Full training** | | | | | | |
| **Task C** | ResNet34 | Class 0 | 0.778(±0.017) | 0.825(±0.021) | | 0.710(±0.020) |
|  |  | Class 1 | 0.500(±0.023) | 0.845(±0.018) | |  |
|  |  | Class 2 | 0.771(±0.017) | 0.897(±0.022) | |  |
|  | GoogLeNet | Class 0 | 0.944(±0.016) | 0.684(±0.020) | | 0.710(±0.021) |
|  |  | Class 1 | 0.500(±0.024) | 0.901(±0.018) | |  |
|  |  | Class 2 | 0.600(±0.023) | 0.966(±0.018) | |  |
|  | MobileNet | Class 0 | 0.861(±0.018) | 0.719(±0.021) | | 0.688(±0.022) |
|  |  | Class 1 | 0.409(±0.023) | 0.916(±0.020) | |  |
|  |  | Class 2 | 0.686(±0.021) | 0.879(±0.020) | |  |
|  | DenseNet | Class 0 | 0.889(±0.018) | 0.737(±0.022) | | 0.677(±0.023) |
|  |  | Class 1 | 0.318(±0.025) | 0.901(±0.019) | |  |
|  |  | Class 2 | 0.686(±0.020) | 0.862(±0.020) | |  |
|  | VGG16 | Class 0 | 0.917(±0.017) | 0.632(±0.022) | | 0.667(±0.020) |
|  |  | Class 1 | 0.917(±0.018) | 0.772(±0.020) | |  |
|  |  | Class 2 | 0.771(±0.019) | 0.845(±0.021) | |  |
| **Doctor diagnoses** | | | | | | |
| **Task C** | Doctor | Class 0 | 0.750(±0.018) | 0.825(±0.022) | | 0.667(±0.021) |
|  |  | Class 1 | 0.474(±0.023) | 0.851(±0.019) | |  |
|  |  | Class 2 | 0.684(±0.019) | 0.818(±0.022) | |  |
| ACC accuracy, SEN sensitivity, SPEC specificity, SD standard deviation | | | | | | |

*Task C: discriminating benign *vs*. borderline *vs*. malignant tumors

*Class 0: malignant tumors; Class 1: borderline tumors; Class 2: benign tumors
